# Can genetic diversity in microalgae species be explained by climate: an overview of metabarcoding with diatoms

**DOI:** 10.1093/ismeco/ycaf171

**Published:** 2025-09-26

**Authors:** Antonija Kulaš, Clarisse Lemonnier, Benjamin Alric, Maria Kahlert, Rosa Trobajo, Marija Gligora Udovič, Frédéric Rimet

**Affiliations:** Faculty of Science, Department of Biology, University of Zagreb, Zagreb HR-10000, Croatia; Université Savoie-Mont Blanc, INRAE, CARRTEL, Thonon-les-Bains F-74200, France; Université Savoie-Mont Blanc, INRAE, CARRTEL, Thonon-les-Bains F-74200, France; Pôle R&D ECLA, INRAE, Thonon-les-Bains, F-74200, France; Department of Aquatic Sciences and Assessment, Swedish University of Agricultural Sciences, Uppsala SE-750 07, Sweden; Marine and Continental Waters, Institute of Agrifood Research and Technology (IRTA), La Ràpita, Catalonia E-43540, Spain; Faculty of Science, Department of Biology, University of Zagreb, Zagreb HR-10000, Croatia; Université Savoie-Mont Blanc, INRAE, CARRTEL, Thonon-les-Bains F-74200, France; Pôle R&D ECLA, INRAE, Thonon-les-Bains, F-74200, France

**Keywords:** cryptic diversity, eDNA metabarcoding, diatoms, allopatric diversification, geographic pattern

## Abstract

Diatoms, a diverse and abundant group of microalgae, play a crucial role in the functioning of rivers, and are widely used as indicators of ecological quality. This microalgae group has an intraspecific genetic diversity that is poorly understood on a global scale. We examined their genetic diversity using metabarcoding data from Nordic to Equatorial rivers (n = 1103 samples). Notably, 61% of genetic variants were endemic to a single climate zone, including 33% from the Equatorial zone. Looking at the genetic diversity within species, one third of the species showed geographic pattern between climate zones and the phylogenetic structure of their communities indicated that they were shaped by environmental filtering. Another third showed no geographic pattern, and their communities were in majority shaped by neutral processes. A final group was between these two situations. Interestingly, no geographic pattern was observed within the same climate zones, even in regions over 10 000 km apart. We conclude that the numerous species showing allopatric diversification between climate zones, would deserve to be separated into new species to improve diatom-based biomonitoring tools. For future studies, expanding geographical sampling coverage, together with using multi-markers or metagenomes approaches would enable to go beyond these results.

## Introduction

Understanding geographic distribution of microorganisms is essential due to their major roles in ecosystems’ functioning [1]. Baas-Becking's hypothesis, “everything is everywhere, but the environment selects”, was, until recently, considered the primary rule for explaining microbial species distributions [2]. It states that most microorganisms exhibit cosmopolitan distributions (e.g. [3, 4]), while others align with a “moderate endemicity model” [5], shaped by species origin, historical dynamics and dispersal limitations (e.g. [6, 7]).

Diatoms are a group of microalgae frequently dominating freshwater biomass, and often used as ecological indicators [8]. Diatom species were previously considered as cosmopolitan due to their small size and large populations [3]. However, recent studies combining molecular and morphological data revealed a huge cryptic diversity [9], challenging species boundaries and distributions [10]. This new insight complicates species identification which until now was based on morphology using their siliceous skeleton, and might change our understanding on diatom species distribution, niche differentiation and evolution. A deeper understanding of factors shaping cryptic diversity in diatoms could improve their use as indicators to monitor ecosystems [11, 12].

Cryptic diversity study in diatoms has mostly been based on cultures, and revealed geographic patterns that suggest possible allopatric speciation [13, 14]. However, culture-based methods are time-consuming and limited to cultivable taxa. In contrast, metabarcoding offers significant potential to study diatoms, revealing high levels of genetic diversity in multiple environmental samples for many species [15]. Using a *rbcL* barcode (a short coding region in RuBisCo gene; [16]), metabarcoding studies showed that most species based on the morphological species concept host genetic variants, with distinct ecological and geographic distributions [17], highlighting a clear benefit for biomonitoring. Across geographically distant areas, the metabarcoding approach showed that almost no genetic variants were shared, indicating high levels of endemism [18]. Even in remote areas with similar climate (e.g. high-altitude alpine lakes [19]), dispersal limitation dominates without any clear geographic pattern, in contrast to the results from earlier culture-based studies [13, 14]. Therefore, extending the application of metabarcoding to a large river dataset in different climate regions offers the opportunity to acquire new insights into the genetic diversity of freshwater diatom species.

Our aim was to assess global genetic diversity within diatoms in rivers and examine its structure across a wide geographic area, guided by two hypotheses.

Firstly, based on evolutionary biology and population genetics (e.g. [20, 21]), we expected that endemic species (restricted to a particular climate zone) would have lower genetic diversity than cosmopolitan species (occurring in at least two climate zones). Additionally, a significant proportion of the genetic diversity would not be identifiable at the species level due to incompleteness in the existing genetic reference barcoding libraries. From this perspective, we predicted that less studied climate zones, such as the Equatorial zone, would have the highest proportion of unknown diversity.

Secondly, as observed on former culture-based studies [13, 14], we expected most diatom species to show a geographic pattern of their genetic diversity within species with clades restricted to specific climate zones. The geographic pattern would suggest that climate and dispersal limitation would play a crucial role in shaping genetic diversity within species. We also hypothesized exceptions among cosmopolitan species known for their rapid dispersal [22]. To explain these diversity patterns, we hypothesized that different ecological processes (deterministic and neutral processes; [23]) would prevail from a species to another.

To address these hypotheses, we analysed diatom communities from rivers across four climate zones (Nordic, Temperate, Equatorial and Mediterranean; adopted from Kottek et al. [24]) spanning Europe, Africa, Central and South America, using environmental DNA (eDNA) metabarcoding approach. To characterize the genetic diversity of the 394 species considered, we used Amplicon Sequence Variants (ASVs) from a 263 bp barcode in the *rbcL* gene. The relative abundance and occurrence of ASVs across climate zones have made it possible to estimate the geographic distribution of species. We assessed clade associations with climate zones for 36 abundant and genetically diverse species using phylogenetic signal tests [25]. Additionally, we evaluated the ecological processes driving the intraspecific diversity of these species by calculating phylogenetic community structure metrics [26].

## Materials and methods

### Study area and sampling procedure

**Figure 1:**
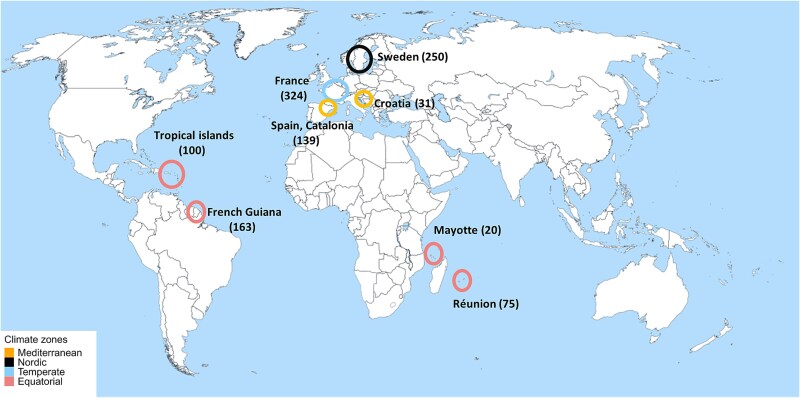
Sampling locations. The circle colours correspond to the respective climate zones, the numbers in brackets indicate the number of samples for each country. Map template created with Canva (www.canva.com).

Through several previous studies, we established a database comprising a total of 1103 samples collected across four climate zones: Nordic, Temperate, Mediterranean and Equatorial from regions in Europe, Central and South America, and Africa (Fig. 1). The significance of these climate zones was assessed by extracting the 19 bioclimatic variables of WorldClim v2 [27] based on the geographical coordinates of the sampling points. A Multi-Response Permutation Procedure was performed on these variables and showed that the differences between climate zones were highly significant (*P* < .001). Mean annual temperatures and precipitation showed marked differences (24°C, 13°C, 10°C, 4°C, and 2200, 699, 950, 657 mm, respectively, for Equatorial, Mediterranean, Temperate, and Nordic). Details of these analyses are given in Supplementary material 1. Samples were collected from 2013 to 2021 (Supplementary material 2) during the stable water period, following a standard protocol [28]. Biofilm was scrubbed from five randomly selected stones, preserved in ethanol with final concentration of 70% (except Croatian samples, stored at −20°C).

### DNA extraction

DNA was extracted from a biofilm pellet, obtained after centrifugation of 2 to 4 ml of the initial biofilm suspension, using soil kits (Supplementary material 2), following the manufacturers' instructions. For the PCR amplification, a short barcode (263 bp) of the *rbcL* chloroplast gene was targeted by using an equimolar mix of three forward primers (Diat_rbcL_708F_1, Diat_rbcL_708F_2, Diat_rbcL_708F_3) and two reverse primers (R3_1, R3_2) [29]. PCR reactions for each DNA sample were performed in triplicate in a final volume of 25 μl following Vasselon et al. [29] procedure. Equimolar concentrations of PCR products (replicates) were pooled for each sample and sent for sequencing to platforms using Illumina MiSeq technology and the v2 reagent kit (2 $\times$ 250 bp), except for the Swedish samples which were sequenced using v3 reagent kit (2 $\times$ 300 bp). All samples were sequenced at the Bordeaux Transcriptome Genome Platform (PGTB, Bordeaux, France), with the exception for Swedish samples which were sequenced at the National Genomic Infrastructure (NGI)/the SNP&SEQ Technology platform (samples in 2020), and at the Swedish University of Agricultural Sciences (samples in 2021) (Supplementary material 2).

### Bioinformatic pipeline

Bioinformatic analyses on the raw data (forward and reverse reads) from the different sequencing runs were launched on the Migale Bioinformatics Facility [30] using the DADA2 pipeline [31] to generate ASVs. Primer sequences were trimmed to forward (R1) and reverse (R2) reads using cutadapt v3.5 [32], and reads were then truncated to 210 (R1) and 130–200 (R2) nucleotides, according to a median quality score ≥ 30 using the *filterAndTrim*() function for the R-package *dada2* [31]. The DADA2 denoising model (*learnErrors*(), *derepFastq*(), and *dada*() function) was used to determine an error model and, finally, *mergePairs*() function was used to merge forward and reverse reads to generate tables of full denoised sequences for each sequencing run. After merging all sequence tables together, chimeras were removed using the *removeBimeraDenovo*() function, and only 263-bp diatom ASVs were retained. Taxonomic assignment was performed using the naïve Bayesian classifier implemented in the R-package *dada2* (*assignTaxonomy*() function), with a minimum bootstrap value of 60% and the ready to use Diat.barcode reference library v.12 for metabarcoding analyses (doi.org/10.57745/XWJJGI) which is an adaptation of the original library [33] trimmed to the 263 bp barcode. After taxonomic assignment, non-diatom ASVs and sequences with stop codons were removed (n = 156, stop codon were detected by converting ASV nucleotide sequences into amino acid sequences using Emboss v6.6.0), bringing the total number of ASVs across the 1103 samples from 12 404 to 12 248. From 12 248 ASVs, 8650 remained after filtering out sequences that could not be assigned at the order level, and further reduced to 3302 ASVs across 1073 samples by removing rare ASVs (fewer than 10 sequences in total) and samples with fewer than 500 reads (Supplementary materials 3 and 4). Ultimately, out of 3302 ASVs, 1397 were not assigned to the species level, while 1905 ASVs corresponded to 394 species. To compare the occurrence of each ASV and species across samples and climate zones, two normalization approaches were applied: rarefaction and Total Sum Scaling (TSS). Following this comparison, the TSS-normalized table was used for downstream analyses, resulting in an ASV relative abundance table with assigned species taxonomy. Full details of the normalization comparison are provided in Supplementary Material 5.

### Statistical analyses

#### Characterization of diatom occurrence patterns

All data analysis and graphical presentations were done using R version 4.3.2 [34].

We first provided an overview of diatom community structure across climate zones with a heatmap based on a normalized (relative abundance) dataset including assigned and unassigned ASVs (3302 ASVs). In the ASV table, samples were organized in rows and ordered in binary format according to the distribution of ASVs. The ASV columns were then sorted based on their incidence across samples using the reciprocal averaging method [35] and the *OrderMatrix*() function from the R package *Metacom* [36].

A Sankey diagram, using the R-package *network3D* [37], illustrated the number of ASVs assigned and not assigned ASVs to species level, those cosmopolitan or endemic, and their distribution across climate zones. Then, we focused on ASVs assigned to species level and examined species distribution based on their abundance, site occupancy, number of ASVs, and climate zone presence.

#### Estimation of geographic patterns of genetic diversity within species and link with climate zones

To evaluate if there was a geographic pattern of genetic diversity within species a subset of species from the initial dataset of the 394 determined species was carried out. The selection was based on ASV counts per species and determined statistically using quantile analysis, resulting in the inclusion of species with at least 10 ASVs, for a total of 36 species (excluding taxa labelled as "*sp*" within a genus; Supplementary material 6). Phylogenies for the 36 selected species were generated from FASTA files using RAxML (version 8.2.12) and R-packages *ape* [38] and *ips* [39]. A maximum likelihood tree was constructed for each species from aligned ASVs sequences, with rapid bootstrap tests and a gamma GTR substitution model based on Modeltest results calculated in raxmlGUI [40].

For each of 36 species, its phylogeny was used to assess the presence of a geographic pattern among its ASVs, using phylogenetic signal tests. We hypothesized that a geographical structure could only be observed between different climate zones, but not between regions belonging to the same climate zone. To this end, we ran two series of tests: the first series focused on ASVs across different climate zones, while the second focused on ASVs within remote regions belonging to the same climate zone. Remote regions belonging to the same climate zone were only available for Mediterranean climate (Spain-Catalonia vs. Croatia, distant by more than 1100 km), and for Equatorial climate (Martinique/Guadeloupe in the West Indies vs. Reunion/Mayotte islands in the Indian Ocean distant by more than 10 000 km, French Guiana was excluded because it presented large continental rivers far different from small insular rivers). To perform the first series of phylogenetic signal test, the species phylogenies for each species were used as input data, along with the number of site occupancies in each climate zone (or remote regions for the second series). For the first series of tests, the number of phylogenetic signal tests was dependent on the number of climate zones in which the ASVs of a given species occurred. For species whose ASVs were present in all four climate zones, four tests (one per climate zone) were performed, whereas only three tests were performed for species with ASVs occurring in three climate zones. For the second series of tests, two tests, one per remote region, were conducted per species in each climate zone, namely Equatorial and Mediterranean. The tests were conducted using the *phyloSignal*() function [41]. For the first series of tests, the number of occupied sites by an ASV was transformed into a frequency for each species within each climate zone (Nordic, Temperate, Mediterranean, and Equatorial). Similarly for the second series of tests, the number of sites occupied by an ASV was transformed into a frequency for each species within each region of a single climate zone (e.g. Spain-Catalonia/Croatia or Guadeloupe/Martinique/Réunion/Mayotte). In order to have robust statistical results, for each species and each series of tests, we ran five different tests: “Cmean” [42]; “I” [43]; “Lambda” [44]; “K” and “K.star” [45]. Since these different tests can give different results for the same input data, we counted the number of significant tests (*P* ≥ 5%) for each species in each climate zone and in each remote region, for the first and second series of tests, respectively. We converted this number into a percentage of significant tests per species and climate zone and per species and remote region, for the first and second series tests, respectively. Then for each species, an average percentage was calculated over the four climate zones for the first series of tests and over the two remote regions for the second series of tests. For each species, these average percentages of significant tests were interpreted as follows: more than 65% significant tests indicate a clear geographic pattern corresponding to phylogenetic signal of ASVs between climate zones (or remote regions for the second series of tests) within the species; 33% to 65% significant tests suggest a weak geographic pattern; less than 33% significant tests reflect the absence of a geographic pattern.

To visualize the distribution of ASVs across climate zones, haplotype networks were constructed for each of the 36 selected species using occupancy data for the respective climate zone. For *Sellaphora nigri* (De Notaris) Wetzel & Ector, a selection of the 69 most frequent ASVs was done (ASVs occurring in less than six samples were discarded). Functions *haplotype*() and *haploNet*() were used under the R-package *pegas* [46].

#### Assessment of ecological processes structuring diatom communities and explaining the co-occurrence of genetic variants

To determine which ecological processes (deterministic and neutral processes) shape the ASVs co-occurrence inside each of the 36 species, the phylogenetic indices Net Relatedness Index (NRI) and Nearest Taxon Index (NTI) were calculated, following Webb et al. [26]. NRI assesses the lineage/phylogenetic distance between all ASVs within a species, while NTI is focused on evaluating pairwise distance between closely related ASVs within a species. Following these definitions, indices were used to measure if ASVs of a species are phylogenetically over-clustered in communities, or are over-dispersed, or do not diverge from null model. For a given species, phylogenetic over-clustering of ASVs suggest that the dominant ecological process in its structuring is environmental filtering. On the contrary, in the case of a phylogenetic over-dispersion, competition dominates, whereas, if indices are neither over-dispersed or over-clustered, they follow a null model. NRI and NTI were already used in former studies to understand assembly rules for diatom species [47, 48]. In addition, the use of phylogenetic distance as a proxy of niche proximity is accepted for diatoms, as there is a conservation of ecological niche through the phylogeny (e.g. [25, 49]). NRI and NTI were calculated with *ses.mpd*(), *ses.mntd*() functions of the R-package *picante* (version 1.8.2) [50] and the phylogenies of each 36 species. For each species, the percentage of NRI and NTI values not significantly different from null model was calculated and used to identify species with or without over-clustering. Species with the lowest ratio of non-significantly different values from null model were considered to have over-clustering, and those with the highest ratio as species with low over-clustering. The similarity in the over-clustering level between the 36 species was illustrated by a heat map was generated using the R-package *pheatmap* [51], with clustering based on an Euclidean distance and the Ward’s sum-of-squares linkage algorithm (Supplementary material 7). A second Sankey diagram was used to illustrate the relationship between the results of the phylogenetic test and phylogenetic clustering for species and their ASVs.

## Results

**Figure 2:**
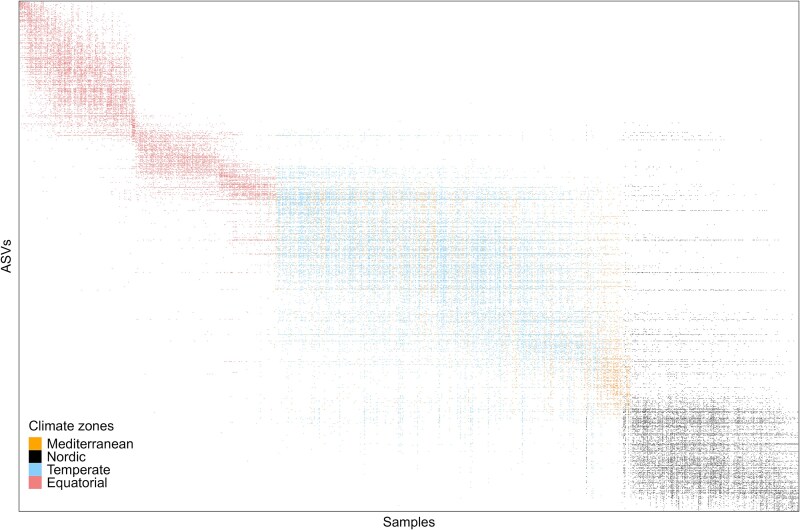
Heatmap of the ASV incidence matrix, after classification by reciprocal mean (Axis 1), showing the distribution of ASVs across the four studied climate zones.

Reorganizing the rows and columns of the global ASV table (1073 samples × 3302 ASVs) using the reciprocal averaging method revealed a distribution pattern of ASVs, along the diagonal of that matrix characterized by the presence of modules associated with climate zones (Fig. 2). However, there were a few exceptions, with some ASVs displaying shared or overlapping distributions, particularly between the Temperate and Mediterranean zones.

### Assessment of unassigned diversity, endemism level, and climate zone’s location of ASVs

**Figure 3:**
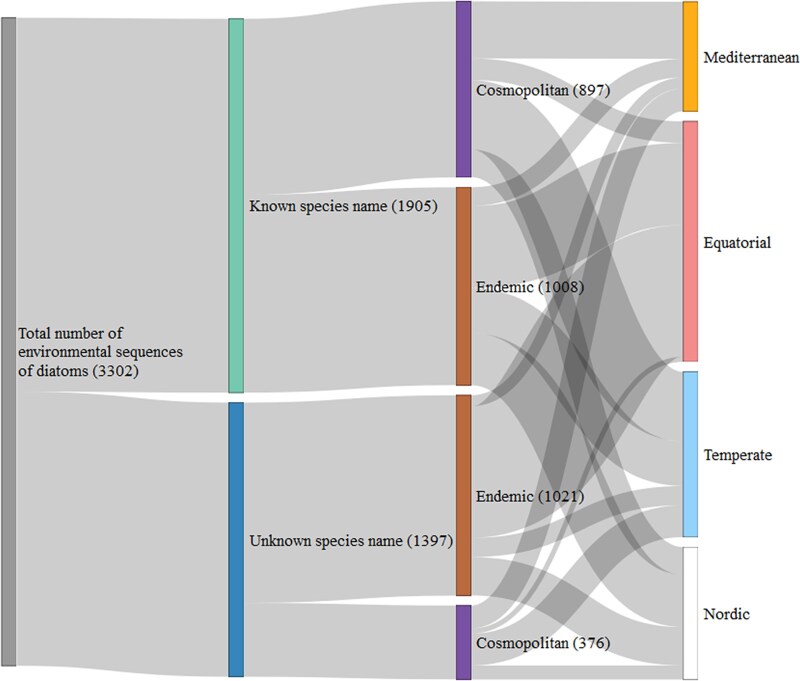
Proportion of environmental sequences (ASVs) whose species name were known or unknown, with their geographical distribution and location in climate zones. Column 1: gives the total number of ASVs. Column 2: ASVs species name were considered as known if it could be assigned using the bioinformatic pipeline and Diat.barcode reference library. Column 3: ASVs were considered endemic if they were present in a single climate zone and cosmopolitan if they were in at least two zones. Column 4: gives the proportion of endemic and cosmopolitan ASV in each climate zone. Numbers in brackets indicate the number of ASVs for each category.

Among the total detected ASVs (n = 3302), 61% were classified as endemic or occurring in only one climate zone, while 39% were cosmopolitan and present in at least two climate zones (Fig. 3). When dividing the dataset into ASVs assigned and unassigned to the species level, 47% of the assigned ASVs were cosmopolitan, compared to 27% of the unassigned ASVs. Notably, within the high percentage of unassigned ASVs detected as endemic, the greatest number of unique ASVs were found in the Equatorial zone (n = 666 ASVs), followed by the Nordic zone (n = 201 ASVs), the Temperate zone (n = 93 ASVs), and the Mediterranean zone (n = 61 ASVs).

### Do cosmopolitan species have greater genetic diversities than endemic species?

**Figure 4:**
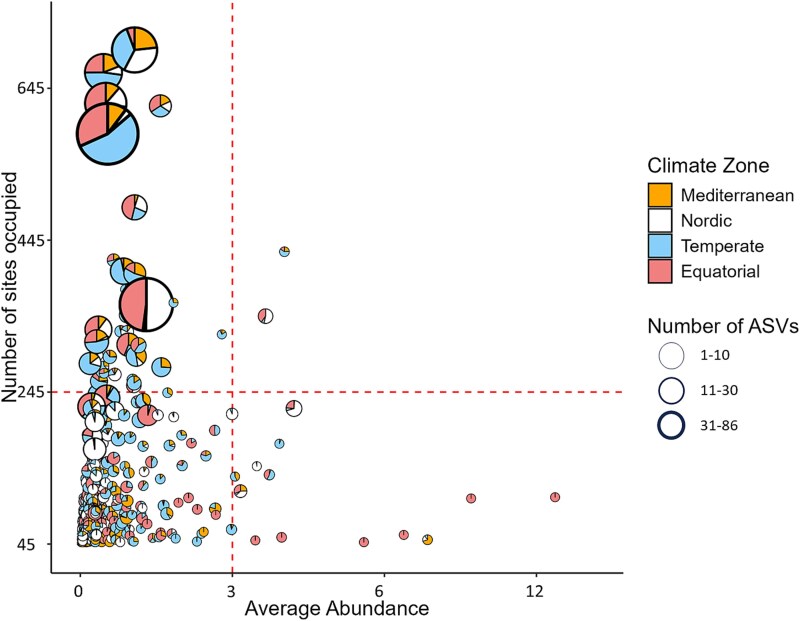
Distribution of diatom species based on their average abundance at a site and their site occupancy (number of sites occupied). The size of each pie chart represents the number of ASVs detected for each species, while the colours of each pie chart indicate the number of sites occupied in each climate zone. The average abundance values represent the average relative abundance per species. Species are differentiated according to the relationship between the sites they occupy and their relative abundance, based on threshold values expressed by the 90th percentiles of occupancy (horizontal line) and average abundance (vertical line).

The analysis of ASVs assigned to the species level (n = 1905) revealed four distinct groups, based on mean values of site occupancy and average abundance (Fig. 4, Supplementary material 6). Each group is differentiated by occurrence and genetic diversity. The top left group corresponded to cosmopolitan species with high genetic diversity (high number of ASVs) and widespread occurrence. This group included *Sellaphora nigri* (De Notaris) Wetzel & Ector, *Eunotia glacialis* F. Meister, *Achnanthidium minutissimum* (Kützing) Czarnecki, *Nitzschia palea* (Kützing) W. Smith, *Navicula cryptotenella* Lange-Bertalot, *Sellaphora pupula* (Kützing) Mereschkovsky, and *Sellaphora saugerresii* (Desmazières) C.E. Wetzel & D.G. Mann. On the opposite side, the bottom right group was represented by endemic species, characterized by a low number of ASVs but high average abundance, and restricted to specific climate zones. This group included species mainly restricted to the Equatorial zone: *Epithemia hirudiniformis* (O. Müll) Rimet, D.G. Mann, R. Trobajo, J. Zimm. & R. Jahn, *Ulnaria goulardii* (Brébisson ex Cleve & Grunow) D.M. Williams, Potapova & C.E. Wetzel, *Epithemia boucheziae* Kochoska, Chardon, Chonova, Keck, Kermarrec, Larras, S.F. Rivera, Tapolczai, Vasselon, Levkov & Rimet, *Halamphora tumida* (Hustedt) Levkov, with the exception of *Stephanodiscus binatus* Håkansson & H.J. Kling, which was not endemic since detected in all zones except Equatorial. The penultimate group, at the bottom left, gathered the largest number of species, characterized by moderate average abundance and number of ASVs, and a wide geographic distribution or restricted to a specific climate zone. The last group, at the top right, included two cosmopolitan species with a low genetic diversity: *Melosira varians* (C. Agardh) and *Eunotia pectinalis* (Kützing) Rabenhorst, present in all climate zones (except Mediterranean for *E. pectinalis*).

### Is there a geographic pattern of genetic diversity inside species and is it related to climate zones?

**Figure 5:**
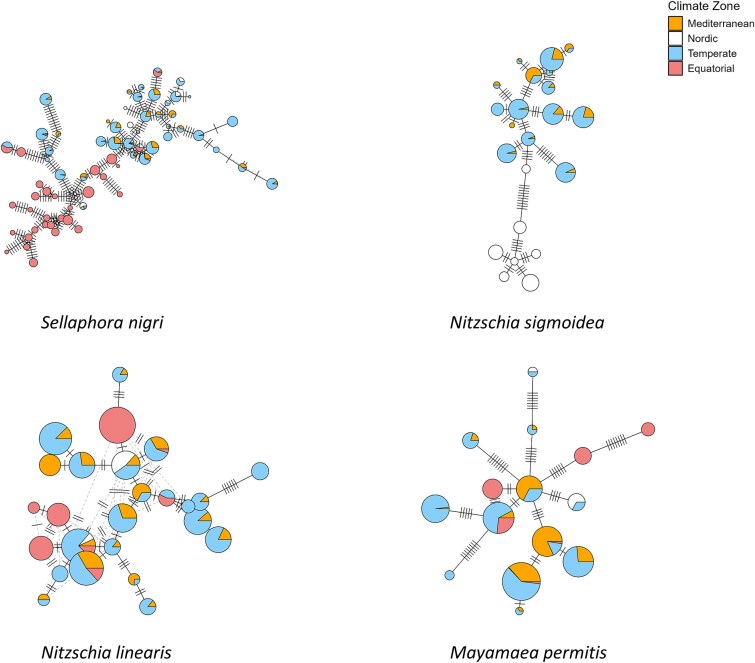
Haplotype network of four diatom species. In a haplotype network, each pie chart corresponds to an ASVs, with the diameter indicating the number of reads in the studied area and the colours indicating its presence in a climate zone. For each edge connecting pie-charts, length and number of lines crossing it correspond to the number of nucleotide differences between ASVs.

Haplotype networks were drawn for 36 diatom species (Fig. 5, Supplementary materials 8 and 9). Among these 36 species, a clear separation by climate zone was observed for instance within *Sellaphora nigri* and *Nitzschia sigmoidea* (Nitzsch) W. Smith (Fig. 5). In contrast, no clear separation depending on climate zone was observed for some species like *Nitzschia linearis* (W. Smith) and *Mayamaea permitis* (Hustedt) K. Bruder & Medlin (Fig. 5). Haplotype networks were consistent with the results of the phylogenetic signal confirming the presence or absence of geographic pattern within species (Supplementary material 10).

**Figure 6:**
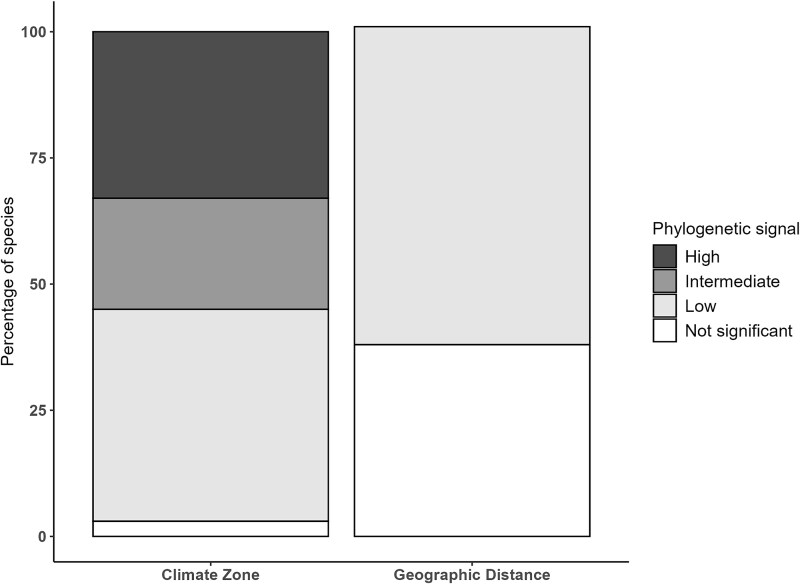
Test of the phylogenetic signal for climate zones (Equatorial, Mediterranean, Nordic, and Temperate) and geographic distance inside a single climate zone (Mediterranean zone: Croatia, Spain-Catalonia; Equatorial: Guadeloupe, Martinique, Mayotte, Réunion islands). Based on an average frequency of five significant tests for each species over the four climate zones and geographic distance within remote regions, species were gathered in four different groups: high phylogenetic signal (average frequency of significant tests ≥0.65), intermediate (0.65 < to <0.33), low (0.33 ≤ to <0) and with no phylogenetic signal (= 0, no significant tests).

Phylogenetic signal levels were compared based on climate zone or geographic distance within remote regions (Fig. 6). Considering the climate zone, 33% of species exhibited a high phylogenetic signal, while 22%, 42% and 3% (only one species) showed an intermediate, low and no phylogenetic signal respectively. In contrast, when assessing the geographic signal within a single climate zone, the majority of species (63%) exhibited a low phylogenetic signal, while the remainders showed no phylogenetic signal at all.

### Which ecological processes dominate within species to explain the co-occurrence of their genetic variants?

**Figure 7:**
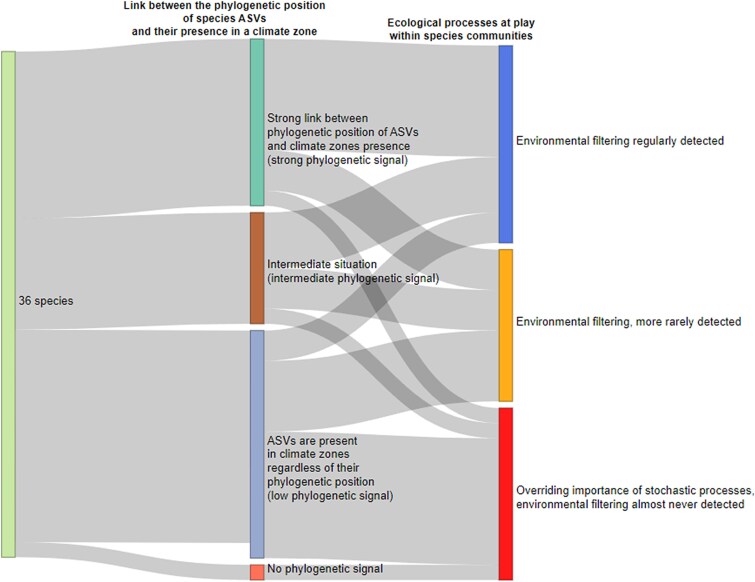
Links between phylogenetic signal of climate zones and ecological processes at play in communities for diatom species commonly observed in rivers. Column 1: indicates the number of species tested. Column 2: indicates the proportion of species with a strong, intermediate, or weak signal based on the three groups of percentages of significant phylogenetic tests per species (high: more than 65% of tests significant, intermediate: between 33% and 65%, low: less than 33%). Column 3: evaluates ecological processes within the phylogenetic indices of species communities. Three categories can be distinguished (from top to bottom). First, phylogenetic over-clustering is regularly detected in species communities (average of 25% to 22%, respectively, for NRI and NTI), indicating a significant impact of environmental filters. Phylogenetic clustering is also detected at lower percentages (average of 10% for NRI and NTI), indicating a lesser importance of environmental filters. Finally, the low percentage of excessive phylogenetic clustering (average of 2% for NRI and NTI) indicates a predominant importance of stochastic processes in the structuring of diatom communities. The results presented in Supplementary Material 7 complement this visualization.

From the same 36 species, the NRI and NTI indices were calculated to determine the ecological processes explaining the occurrence of their respective ASVs (Supplementary materials 7 and 10). All NRI and NTI were positive, indicating an over-clustering, with 19% being statistically significant (*P* < .05). A cluster analysis based on NRI and NTI results identified four groups (according the truncation threshold; Supplementary material 7) that, for simplicity’s sake, can be grouped into three groups. The first group, which includes two, comprised 14 species (e.g. *Sellaphora nigri*, *E. glacialis*) showing strong phylogenetic over-clustering within species communities (ratio of values not significantly different to null model ranging from 0.65 to 0.80). The second group of 11 species (e.g. *Achnanthidium pyrenaicum* [Hustedt] H.Kobayasi*, E. pectinalis* [Kützing] Rabenhorst) exhibited weaker over-clustering (ratio of values not significantly different to null model ranging from 0.80 to 0.95). The last group comprised 11 species (e.g. *Staurosira construens* Ehrenberg, *Nitzschia fonticola* (Grunow) Grunow) that showed a low over-clustering with a dominance of the null model (ratio ranging from 0.95 to 1). When comparing phylogenetic signal with NRI and NTI indices, 67% of species with a high phylogenetic signal demonstrated strong environmental filtering or over-clustering, while 56% of species with a low phylogenetic signal did not diverge from null model (Fig. 7).

## Discussion

This extensive eDNA metabarcoding dataset enabled us to analyse the genetic diversity of diatom species across four distinct climate zones spanning four continents. Our study revealed that genetic variants (ASVs) within diatom species are often region-specific, emphasizing the critical role of genetic resolution in defining species boundaries. This aligns with Mann's [52] species concept that the definition of diatom species should reflect their true diversity, beyond morphology alone. Although species concepts remain widely debated, metabarcoding and the taxonomic assignment of ASVs through a given barcoding library (here Diat.barcode) provide a consistent and standardized method for species identification and genetic diversity assessment. In this respect, diatom intercalibration exercises, which highlight the value of a unified approach over individual analyst expertise [53], show the importance of harmonized diatom identification. Metabarcoding thus improves species classification, by offering a more objective alternative to traditional morphological method (e.g. reducing reliance on the expertise of individual analysts; [54], and shifting the focus from the taxonomic resolution itself to ASVs captured by this resolution [55].

### A large proportion of the diversity is not identified at species level and is endemic of the Equatorial zone

Across the dataset (including ASVs assigned and not assigned to a taxonomic level such as species) endemic taxa (61%) consistently outnumbered cosmopolitan taxa (39%), especially among ASVs not assigned to species level. Another interesting point is that Equatorial zone presented the highest percentage of endemic taxa (33%), with also a predominance of ASVs not assigned to the species level. This result supports our first hypothesis, emphasizing that there is still a great undiscovered diversity in the Equatorial region which is therefore not referenced, and this aligns with Mann and Vanormelingen [56] who believe that these regions are under-studied. Additionally, the majority of ASVs were specific to a single climate zone. The high endemism of the Equatorial zone is mostly explained by French Guiana (South America), which is consistent with the hotspots of diatom diversity already recognized and high endemism in Amazonia [57]. Tropical islands, on the other hand, have a lower rate of endemism than continental areas [18] making them moderate contributors to the global Equatorial endemism. This suggests that, although island isolation may promote endemism, the unique and environmentally heterogeneous habitats of continental regions appear to be more important drivers of localized diversity patterns in the Equatorial zone [58]. Interestingly, Nordic climate zones host also a high number of endemic taxa. In contrast, the Temperate and Mediterranean zones have the lowest endemic diversity, likely due to their close geographic proximity in Europe, which facilitates dispersal. Overall, these findings emphasize the importance of targeted sampling in underexplored regions, in order to fully capture the extent of diatom diversity and understand the driving factors in species endemism.

### Cosmopolitan species have greater genetic diversities than endemic species, with a few exceptions

A positive relationship was found between species site occupancy and genetic diversity, with species found in multiple sites exhibiting higher genetic diversity, such as *A. minutissimum*, *N. palea*, *N. cryptotenella*, *Sellaphora nigri*, *S. pupula*, and *S. saugerresii*, which are recognized as cosmopolitan species [59]. This pattern is consistent with observations in other organisms, including plants (e.g. [60, 61]) and bacteria (e.g. [55, 62]). Cosmopolitan species tend to have greater evolutionary flexibility and thus greater genetic diversity [63], which enables them to thrive in diverse environments and occupy a wide range of ecological niches. On the other hand, we observed several endemic diatom species restricted to the Equatorial zone, including *Epithemia hirudiniformis*, *E. boucheziae*, *Ulnaria goulardii*, and *Halamphora tumida*, which is consistent with previous findings in tropical areas [64–66]. Among these, *H. tumida*, initially described as *Amphora tumida* Hustedt from Venezuela (South America), is a special case, since it is in line with our findings, being detected exclusively in the Equatorial zone, whereas subsequent studies have reported this species in widespread habitats [67]. It should be noted, however, that in these studies, *H. tumida* was identified solely on the basis of morphological characteristics [67], and may not correspond to the same species. Endemic species typically show lower genetic diversity due to their restriction to a specific location and limited geographic distribution [60]. Despite the general trend of genetically diverse taxa being cosmopolitan and genetically poor taxa being endemic, exceptions were observed in three examples. Cosmopolitan species, such as *M. varians*, with high abundance across all four climate zones, exhibited a low genetic diversity, as observed previously by Zetzsche et al. [68]. Similarly, *E. pectinalis* and *Stephanodiscus binatus*, detected in three climate zones are also usually recognized as a cosmopolitan species [69, 70], but also presented with poor genetic diversity. Although, our dataset was primarily composed of benthic taxa, we acknowledge that planktonic diatoms (*S. binatus* or *M. varians*) may follow different biogeographic and evolutionary patterns. It has been shown that ecological traits like lifestyle (benthic vs. planktonic) can influence dispersal, with some studies suggesting a greater dispersal capacity in planktonic species [22], while others found no significant effect [71]. This remains a hypothesis at the global scale that was not directly tested here. Previous studies (e.g. [9, 15]) on diatom evolution showed that diversification rates vary between genomes and genes across different genera and species. While some genes may work well for identifying species within a genus, they can be too conserved for others [9]. The low genetic diversity observed in some species may be due to the use of a single gene marker, *rbcL,* and using multi-markers or metagenome-assembled genomes approaches would provide additional insights.

### For a majority of the species, genetically similar ASVs are found in the same climate zone

Phylogenetic signal tests showed that there was a geographical pattern (i.e. significant phylogenetic signal) for a majority of species, for which the genetically similar ASVs were located in the same climate zone, with the nuance that the geographical pattern was strong for 33% of species and intermediate for 22% of species. These results, obtained from environmental data on a broad geographical scale, extend the findings of previous studies based on cell culture showing that within certain species, phylogenetic clades were restricted to geographical areas. This is the case for *S. pupula*, for which dozens of cultures were sequenced, and for which the phylogenetic cluster and distribution in defined geographical zones whereas clearly established [13, 72]. Similarly, *Gomphonema parvulum* and *Pinnularia borealis* cell cultures, exhibited clades restricted to different climate zones [9, 14]. For these species, the authors concluded that ongoing speciation or that different species were present. Interestingly, according to a study based on culture method [73], a complex pattern of genetic and physiological variation tied to specific environmental conditions was also observed for *Nitzschia inconspicua*, whereas in a field study [17] no significant differentiation was found in the ecological preferences of ASVs identified by a metabarcoding approach. This discrepancy between the results of culture and eDNA metabarcoding highlights how methodology can influence observed patterns; with cultures isolating specific ecotypes and eDNA metabarcoding capturing broader natural diversity. For *A. minutissimum*, the previously cited study [17] also found that ASVs formed distinct grouping according to their ecological preferences. In our study, both of these species (*N. inconspicua* and *A. minutissimum*) exhibited a high phylogenetic signal within climate zones, supporting the role of local environmental filters and climate to shape their intraspecific diversity.

On the other hand, previous research suggested other factors shaping intraspecific genetic diversity, which could explain the weaker phylogenetic signal observed in 44% of species. Notably, Vanelslander et al. [74] showed that genetic divergence can arise from ecophysiological adaptations of individual living in sympatry, facilitating niche partitioning among closely related species. Such a process could explain the weaker phylogenetic signal we detected for *N. linearis*. In our study, most of the *N. linearis* ASVs appeared in both Mediterranean and Temperate zones. Minimal geographic isolation may have facilitated sympatric diversification in *N. linearis*. Reproductive isolation could have led to divergence in another examples, such as *Eunotia bilunaris*, as shown by Vanormelingen et al. [59]. Sympatric diversification inside species with weaker phylogenetic signal may also be explained by other processes such as intraspecific competition, influenced by resource availability like nutrients and light [75].

Overall, our results highlight that for a majority of the species (55%), clades of ASVs diverge between different climate zones, which may suggest the presence of ongoing allopatric diversification or different species. In addition, we observed that when considering different geographical regions within the same climate zone, no link can be established between genetic similarity of ASVs and their geographic origin. For example, even with several thousand kilometres between geographical regions within the same climatic zone (e.g. Equatorial zone), geographical distance does not appear to shape intraspecific genetic diversity.

### Which ecological processes dominate within species to explain the co-occurrence of their ASVs?

For species with a strong phylogenetic signal, most of their co-occurring ASVs were closer phylogenetically than expected from null model, suggesting that environmental filtering is a predominant process. According to Webb et al. [76], closely related species with shared physiological tolerances tend to co-occur under strong environmental filtering when relevant traits are conserved [24]. This supports that environmental conditions could shape species distributions across different climate zones in our study. Such findings align with the theoretical framework suggesting that taxonomically related groups with similar ecological niches can persist under favourable environmental conditions [76]. For example temperature and light play a significant role in intraspecific competition among diatom sub-species, in aquatic ecosystems [77]. Consequently, the greater temperature variability in Temperate region than in Equatorial region, where conditions are more stable [24], may act as an environmental filter strongly influencing the distribution of intraspecific diatom diversity. It is important to consider that the geographic separation of studied climate zones combined with the large-scale atmospheric circulation patterns, like Hadley, Ferrel, and Polar cells, may further influence species dispersal [78]. These atmospheric patterns, which determine wind directions, temperature gradients, and moisture distribution, can act as barriers to species movement, particularly for species dependent on stable environmental conditions, limiting their ability to move easily across different climate regions [78, 79]. Conversely, for most of species showing no link between ASVs similarity and climate origin (weak or no phylogenetic signal), co-occurring ASVs were not phylogenetically closer than expected by random (i.e. null model). This indicates the greatest importance of neutral processes such as dispersal and ecological drift in line with neutral theory's assumption of ecological equivalence where environmental preferences are not strongly limiting [80]. It is essential to consider that dispersal ability in microbial communities, including diatoms, can vary also due to deterministic traits such as spore formation, morphological adaptations, and habitat specificity as well as physiological traits that determine tolerance to environmental fluctuations [59]. Indeed, such deterministic adaptations can facilitate widespread dispersal, leading to more uniform distributions and increasing the apparent influence of neutral processes in structuring their communities, a context-dependent interplay between selection and stochastic forces consistent with Vellend's conceptual synthesis [81]. Both neutral and deterministic factors, including habitat features and seasonal variation in favourable conditions, likely interact to influence dispersal success and the establishment of species in new habitats [55].

In conclusion, our results revealed that 61% of ASVs were endemic to a specific climate zone, with 33% of them occurring in the Equatorial zone. Additionally, for 55% of species, ASVs showed diversification patterns linked to climate zones, potentially leading to allopatric speciation. However, these results must be considered in light of certain limitations of our study. The geographical sampling effort was biased toward the Equatorial zone, which has many island samples and was more widely distributed spatially than Temperate, Mediterranean and Nordic zones. An improvement would be to include more continental regions for each climate zone in the future. We recommend taking advantage of nucleotide archive platforms (e.g. European Nucleotide Archive, Sequence Read Archive) where datasets from various geographic origins (e.g. America, Asia, Africa) using the same *rbcL* barcode have recently been deposited. We also encourage people using the same *rbcL* barcode which would increase data coverage and enhance comparability across regions. During this study, we relied on a short region of a single genetic marker. Although *rbcL* has demonstrated several advantages over other gene markers commonly used for diatoms (18S, cox1, ITS; [13]), it would be interesting to go beyond the use of one or more short fragments and consider the use of long fragment using long-read technology (e.g. Oxford Nanopore Technology, Pacific Biosciences), or metagenomes with shot-gun sequencing in order to access more accurate information on genetic diversity. Finally, data on local factors, such as physico-chemical parameters, were rarely available due to differences in the protocols used, the instruments employed, and data sharing between laboratories. We circumvented this issue by using a null model-based approach (NTI and NRI indices) to estimate the ecological processes (deterministic and neutral processes) that drove the structuring of diatom communities. In the future, integrating both climatic and parameters *in-situ* measured into such a study will provide a more comprehensive understanding of the local factors driving community assembly. Despite the limitations listed above, our study provides new insights on the cosmopolitan or endemic nature of diatom species and on the ecological processes driving their distribution. It also offers new perspectives for assessing ecological quality of aquatic ecosystems under global change. For example, our results could bring improvement of diatom-based biomonitoring tools. Species exhibiting diversification/speciation across different climate zones deserve particular attention, as they occupy distinct geographical locations, thrive in varied environments, and belong to different phylogenetic clades. From a taxonomic point of view, consideration should be given to separating these species into new species. This would strengthen the use of diatoms as ecological indicators, especially since biotic indices are highly dependent on taxonomic accuracy [82]. Additionally, many of the species with high phylogenetic signal and allopatric diversification in our study were small cell-sized species, which are difficult to identify using traditional morphological methods (e.g. light microscopy). This reinforces the advantages of eDNA metabarcoding for detecting cryptic diversity in biomonitoring analyses.

## Supplementary Material

Supplementary_material_1_ycaf171

Supplementary_material_2_ycaf171

Supplementary_material_3_ycaf171

Supplementary_material_4_ycaf171

Supplementary_material_5_ycaf171

Supplementary_material_6_ycaf171

Supplementary_material_7_ycaf171

Supplementary_material_8_ycaf171

Supplementary_material_9_ycaf171

Supplementary_material_10_ycaf171

Kulas_et_al_supplementary_information_ycaf171

## Data Availability

All data needed to evaluate the conclusions in the paper are present in the paper or in the Supplementary Information. The final results of the bioinformatics analysis, including DNA sequences, are provided in Supplementary materials 3 and 4. R scripts used for processing data for the entire analysis are available as a GitHub project deposited at https://github.com/tonkakulas/diatoms_cryptic_diversity.git.
