# Correction to: Can genetic diversity in microalgae species be explained by climate: an overview of metabarcoding with diatoms

**DOI:** 10.1093/ismeco/ycaf236

**Published:** 2026-03-18

This is a correction to: Antonija Kulaš, Clarisse Lemonnier, Benjamin Alric, Maria Kahlert, Rosa Trobajo, Marija Gligora Udovič, Frédéric Rimet, Can genetic diversity in microalgae species be explained by climate: an overview of metabarcoding with diatoms, *ISME Communications*, Volume 5, Issue 1, January 2025, https://doi.org/10.1093/ismeco/ycaf171

The following changes have been made to the originally published manuscript.

Detail in the affiliation of the fifth author has been emended to read: “[...]Catalonia[...]”.

Under heading **Do cosmopolitan species have greater genetic diversities than endemic species?**, first paragraph, the sixth sentence is emended to read: “This group included[...]” instead of: “This group includes[...]”.

Under heading **Which ecological processes dominate within species to explain the co-occurrence of their genetic variants?** text in the sixth sentence is corrected to read: “The last group comprised 11 species (e.g. *Staurosira construens*[...]” instead of: “The last group comprised 11 species (e.g. *Staurosia construens*[...]”.

Only the first of ten Supplementary files was published: files 2-10 are now added to the article.

