# Supplementary material for: Can genetic diversity in microalgae species be explained by climate: an overview of metabarcoding with diatoms
Source: ISME Commun. 2025 Sep 26;5(1):ycaf171. doi: 10.1093/ismeco/ycaf171 (PMC12527276; doi:10.1093/ismeco/ycaf171)
Supplement: Supplementary_material_1_ycaf171 [file supplementary_material_1_ycaf171.pdf]

Supplementary material 1 - Climate zone characterisation and evaluation of their robustness for the sampled sites

To characterize the four climate zones we used WorldClim dataset (Fick and Hijmans, 2017) available at https://www.worldclim.org/ . Bioclimatic variables were downloaded (10 minutes dataset).

All manipulation were carried out in R. The data of the 19 bioclimatic variables (WorldClim v2) were extracted based on the geographical coordinates of each sample using the extract function of terra package. Here is a map showing the sampling sites on the annual average temperature map (Fig. S1).

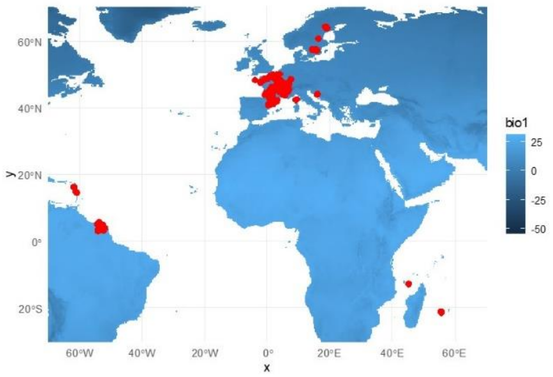

Figure S1. Location of the sampling sites on the annual average temperature map.

Then after normalising the data, a NMDS was calculated with the *metaMDS*() function of *Vegan* package using Euclidian distance. MDS results are given in the figure below (Fig. S2).

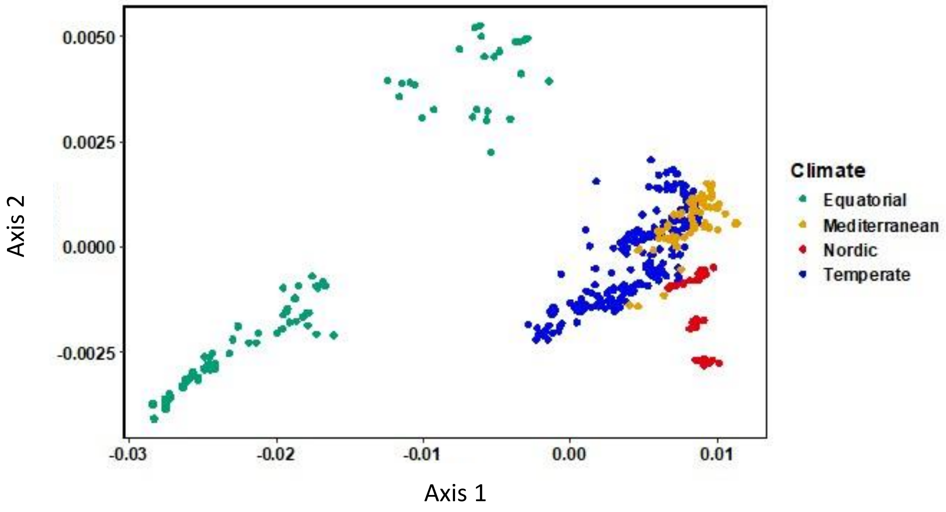

Figure S2. NMDS based on the 19 bioclimatic variables, for the sampling sites of our study

A multi-response permutation procedure (MRPP) was run with 999 permutation with *mrpp* () function in *Vegan* package and gave very significant results for the climate zones (*p*-value <0.0001). Therefore, we consider that climate zones defined are meaningful for our study, even if an overlap between Mediterranean and Temperate zones.

The table (Table S1) below gives for a selection of parameters the averages calculated on the data extracted for the sampling points.

Table S1. Averages in each climate zone of a selection of WorldClim variables. Averages were calculated with the data extracted for the sampling points of our study. Isothermality is the ratio between Mean Diurnal Range and Temperature Annual Range, Precipitation Seasonality is the coefficient of variation of precipitation.

**Climate zone**	**Annual Mean Temperature (°C)**	**Isothermality**	**Annual Precipitation (mm)**	**Precipitation Seasonality**
Equatorial	24,3	69,5	2200,0	47,7
Mediterranean	13,3	37,2	699,6	27,1
Nordic	4,2	29,0	657,0	27,7
Temperate	10,0	35,1	950,2	15,5

Reference Fick SE and Hijmans RJ. WorldClim 2: new 1km spatial resolution climate surfaces for global land areas. *Int J Climatol* 2017; **37**(12): 4302-4315.
