# Supplementary material for: Can genetic diversity in microalgae species be explained by climate: an overview of metabarcoding with diatoms
Source: ISME Commun. 2025 Sep 26;5(1):ycaf171. doi: 10.1093/ismeco/ycaf171 (PMC12527276; doi:10.1093/ismeco/ycaf171)
Supplement: Supplementary_material_5_ycaf171 [file supplementary_material_5_ycaf171.pdf]

**Supplementary material 5 -** ASV and Species Distribution Across Climate Zones: Rarefaction vs. TSS

To assess the robustness of our findings, we performed rarefaction to the minimum sequencing depth (5,625 reads; Fig. S3) and compared results obtained from rarefied and TSS (Total Sum Scaling) normalized data. In Fig. S4a and S4b, we show this comparison using the same data structure as in Fig. 3 of the manuscript, which displays the proportion of environmental sequences (ASVs) of diatoms with known and unknown species names, along with their geographic distribution across climate zones.

Additionally, we assessed the occurrence of each ASV and species across samples and climate zones using both normalization approaches. We defined endemic taxa as those present exclusively in a single climate zone and compared their total abundance and frequency of occurrence with those of cosmopolitan taxa (found in multiple zones). To test whether apparent endemism could be explained by undersampling, we used the Wilcoxon rank-sum test to compare total abundance between endemic and cosmopolitan taxa. The test was run using the *wilcox.test*() function in R, with Bonferroni correction applied to account for multiple comparisons. This approach was chosen due to the non-normal distribution of the data and the presence of unequal variances between groups. Each taxon was treated as a single data point, using its total abundance across all samples as the test variable. The test revealed highly significant differences in both datasets (rarefied: $p < 2.2x\ 10^{-16}$; TSS: $p = 1.1x\ 10^{-12}$). These results indicate that endemic taxa are not simply less common due to sampling noise but differ consistently from cosmopolitan taxa in abundance and distribution. For all analysis in the MS we used TSS normalized data (Wang et al., 2024).

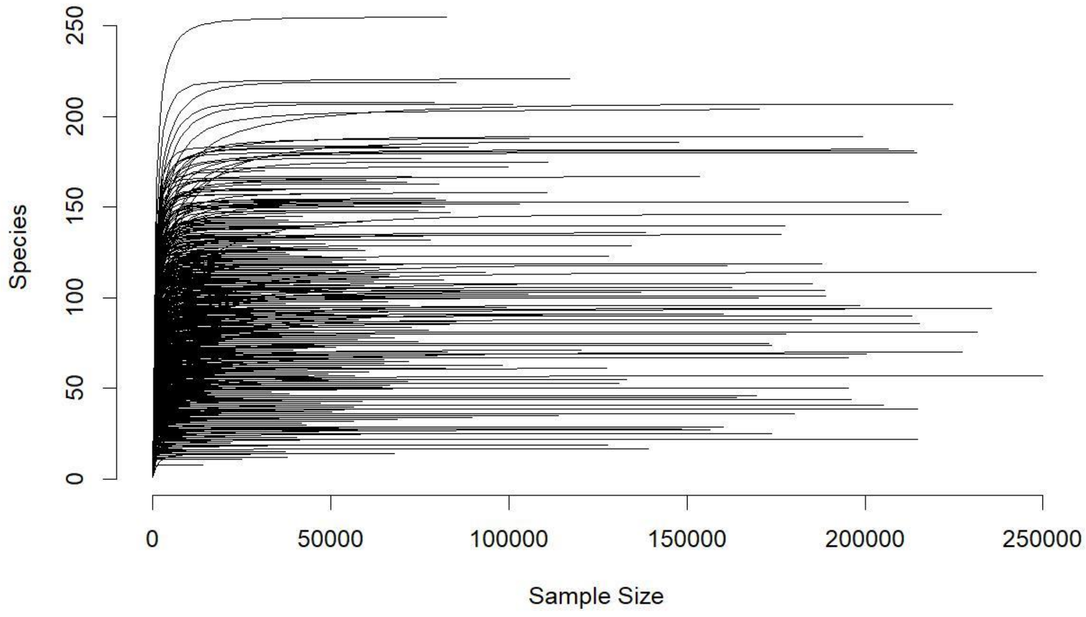


**Figure S3.** Rarefaction plot. The smallest sample size after quality filtering was: 5,625.

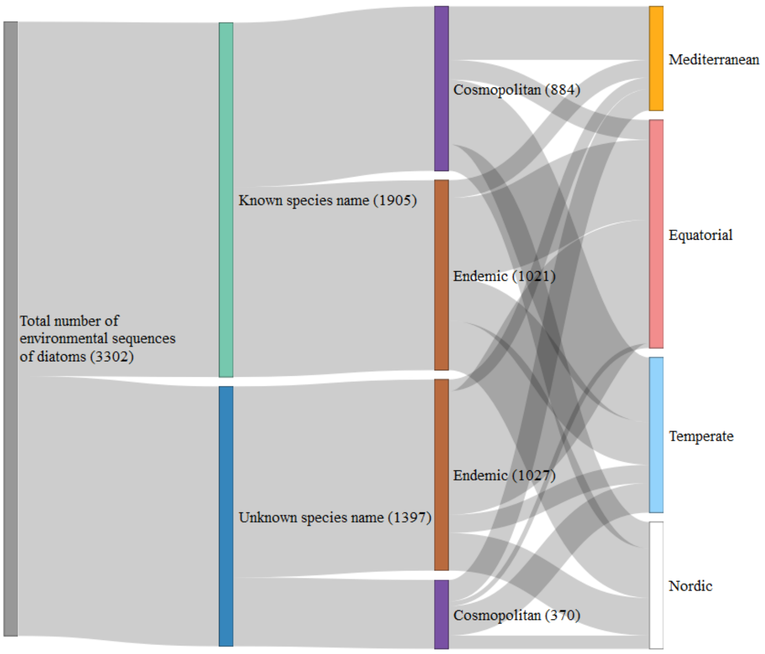


**Figure S4. a)** Sankey diagram with rarefied data (Proportion of environmental sequences (ASVs) of diatoms whose species name were known or unknown, with their geographical distribution and location in climate zones).

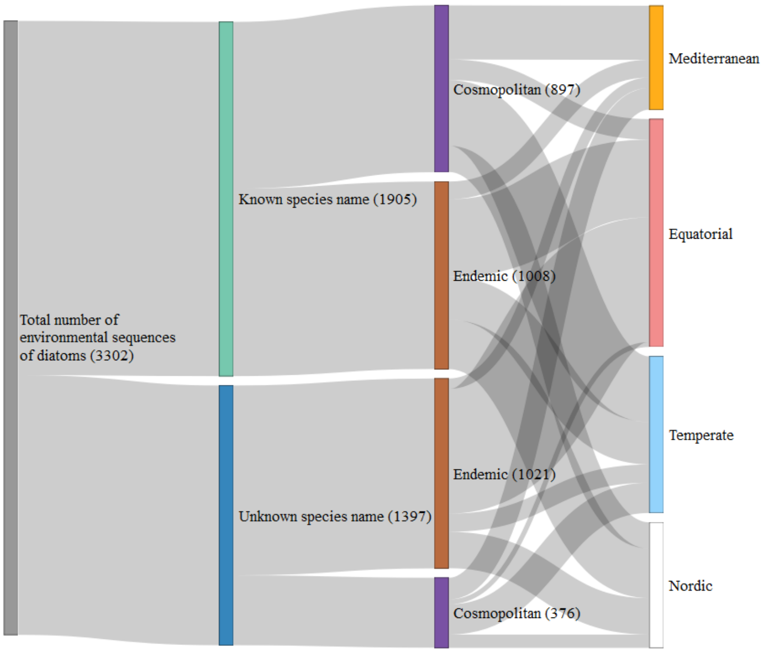


**Figure S4. b)** Sankey diagram with TSS normalized data (Proportion of environmental sequences (ASVs) of diatoms whose species name were known or unknown, with their geographical distribution and location in climate zones), presented in the MS as Fig. 3.

**Reference:** Wang Z, Lloyd D, Zhao S, Reif-Motsinger A. Taxanorm: a novel taxa-specific normalization approach for microbiome data. *BMC Bioinformatics* 2024; **25**: 304. https://doi.org/10.1186/s12859-024-05918-z
