# Supplementary material for: Can genetic diversity in microalgae species be explained by climate: an overview of metabarcoding with diatoms
Source: ISME Commun. 2025 Sep 26;5(1):ycaf171. doi: 10.1093/ismeco/ycaf171 (PMC12527276; doi:10.1093/ismeco/ycaf171)
Supplement: Supplementary_material_7_ycaf171 [file supplementary_material_7_ycaf171.pdf]

Supplementary material 7 - Heatmap of null model ratio for phylogenetic indices NRI and NTI for 36 species

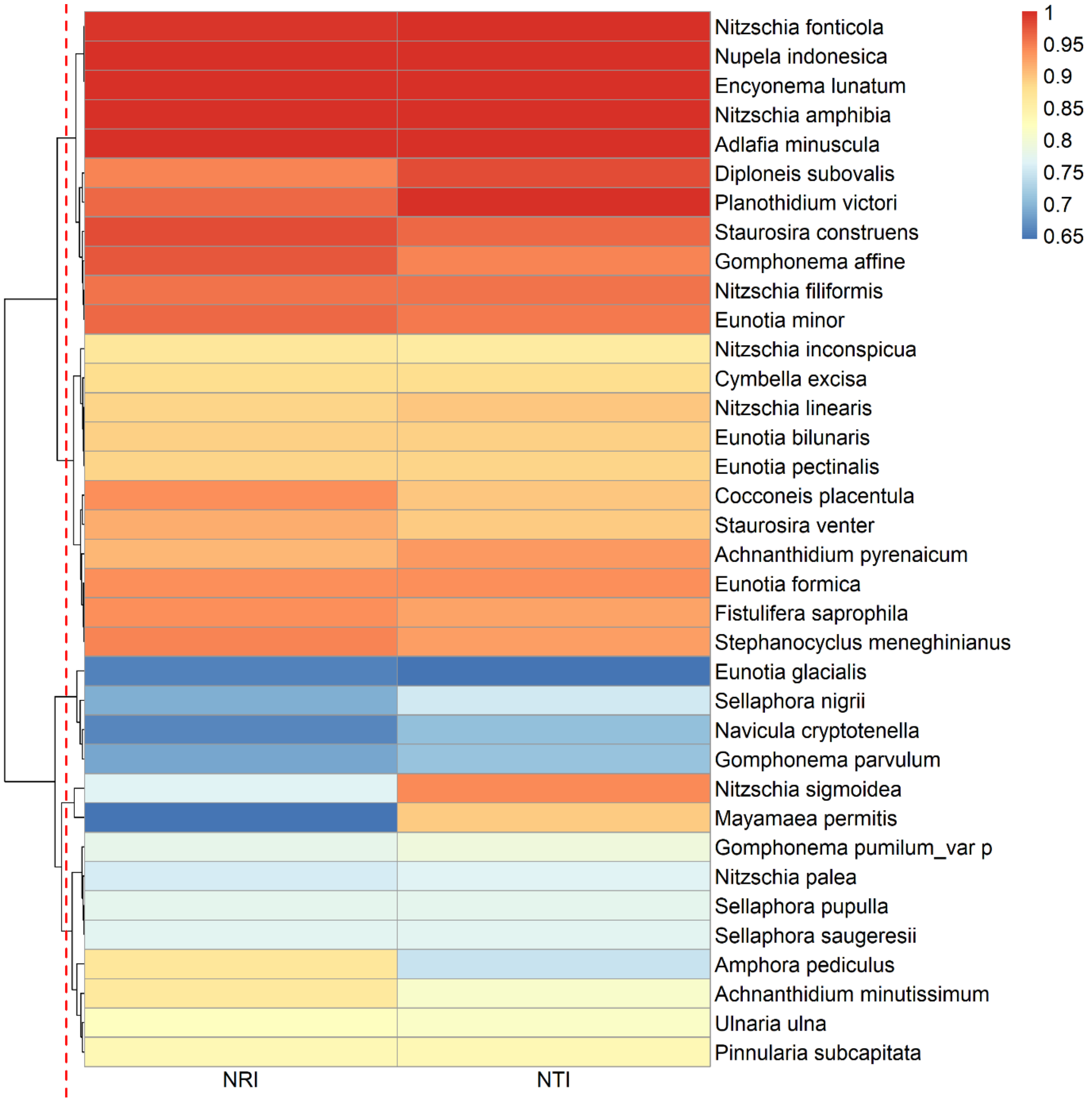

Figure S5. Heatmap of the number of values not significantly different from a null model ratio for phylogenetic indices NRI and NTI for the 36 species. A Euclidean distance was used to compute the dissimilarity distance between the 36 diatom species based on the NRI and NTI indices. The resulting distance matrix was then used in a hierachical cluster analysis with Ward's sum-of-squares linkage algorithm. Label *Gomphonema pumilum_var p* corresponds to species name *Gomphonema pumilum var. pumilum* Grunow.

For simplicity's sake interpretation was that species were clustered into 3 groups based on the percentage of indices values (NRI/NTI) non-significantly different from a null-model: these 3 groups corresponded to strong (from 65 to 80% of NRI-NTI values not significantly different from null-model); intermediate (from 80 to 95%) and low (from 95 to 100%) environmental filtering/over-clustering. This visualization complements Sankey diagram presented at Fig. 7 in MS.
