# Supplementary material for: Can genetic diversity in microalgae species be explained by climate: an overview of metabarcoding with diatoms
Source: ISME Commun. 2025 Sep 26;5(1):ycaf171. doi: 10.1093/ismeco/ycaf171 (PMC12527276; doi:10.1093/ismeco/ycaf171)
Supplement: Supplementary_material_8_ycaf171 [file supplementary_material_8_ycaf171.pdf]

Supplementary material 8 - Haplotype network of other 32 species

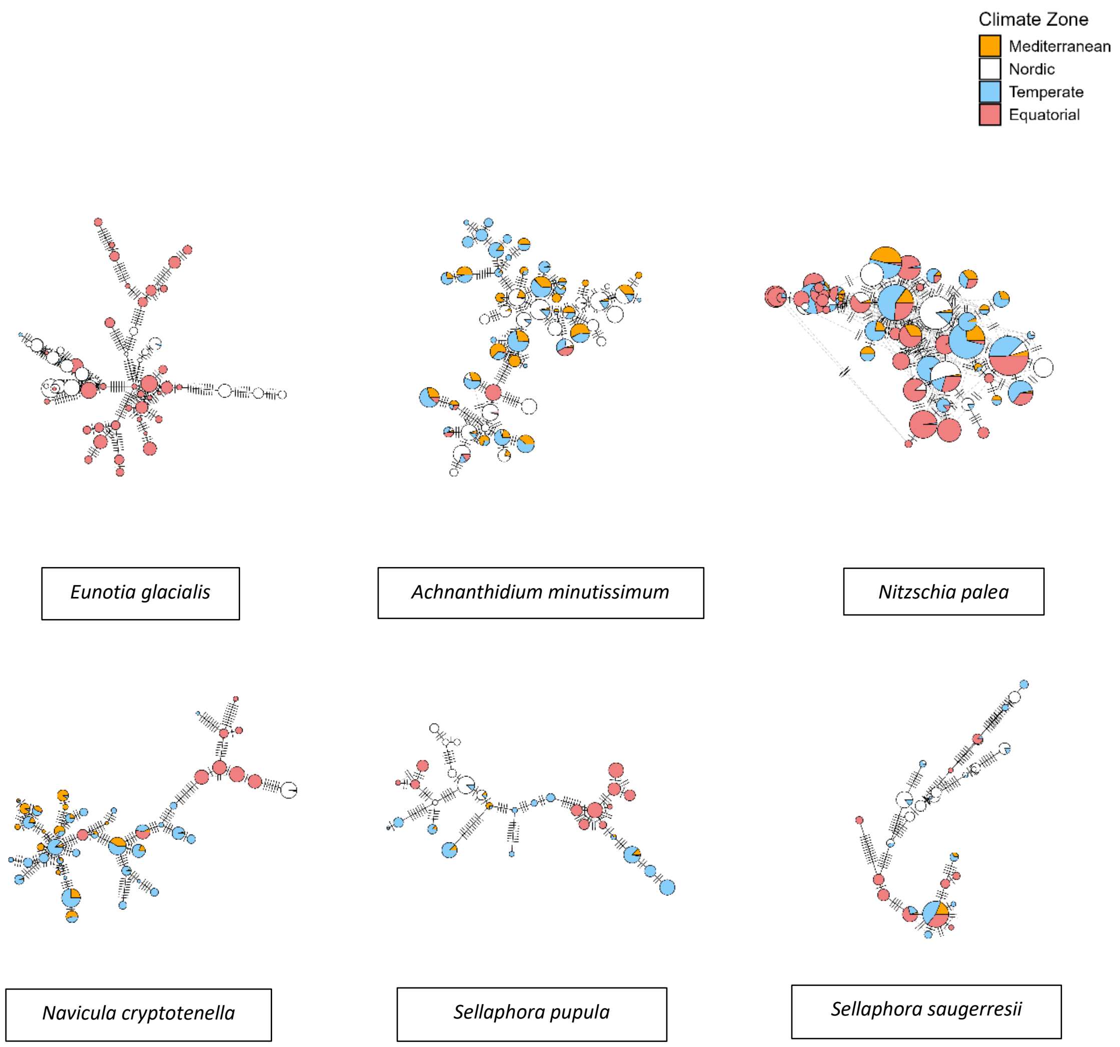

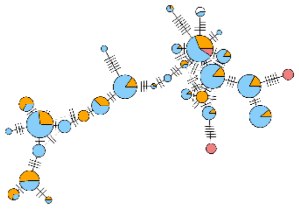

Amphora pediculus

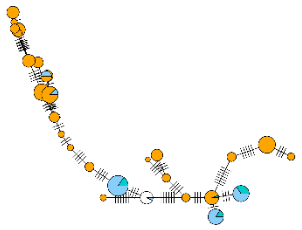

Nitzschia filiformis

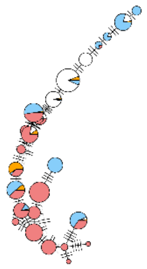

Gomphonema parvulum

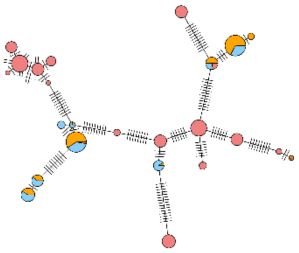

Nitzchia incospicua

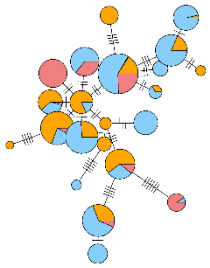

Fistulifera saprophila

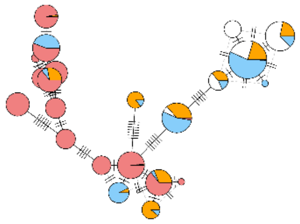

Ulnaria ulna

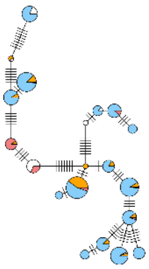

Cocconeis placentula

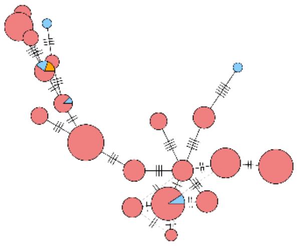

Gomphonema affine

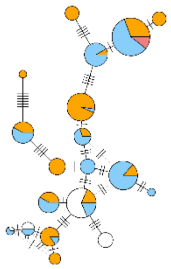

Cymbella excisa

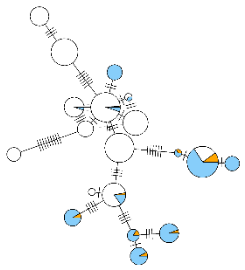

Staurosira venter

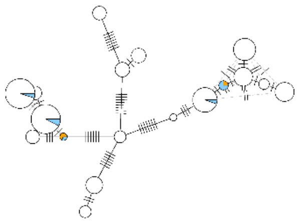

Eunotia bilunaris

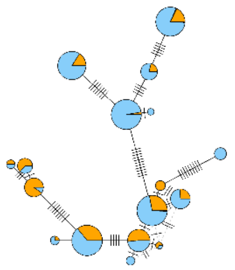

Achnanthidium pyrenaicum

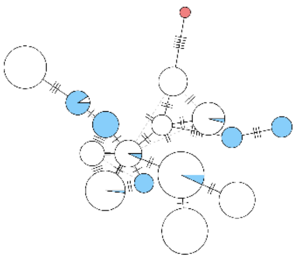

Eunotia minor

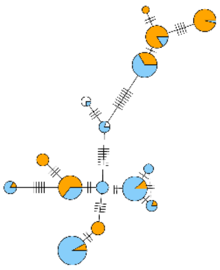

Diploneis subovalis

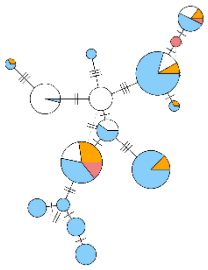

Staurosira construens

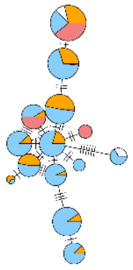

Stephanocyclus meneghinianus

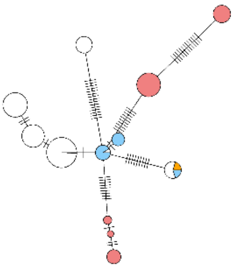

Eunotia formica

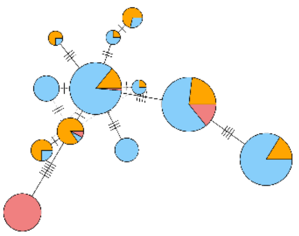

Nitzschia fonticola

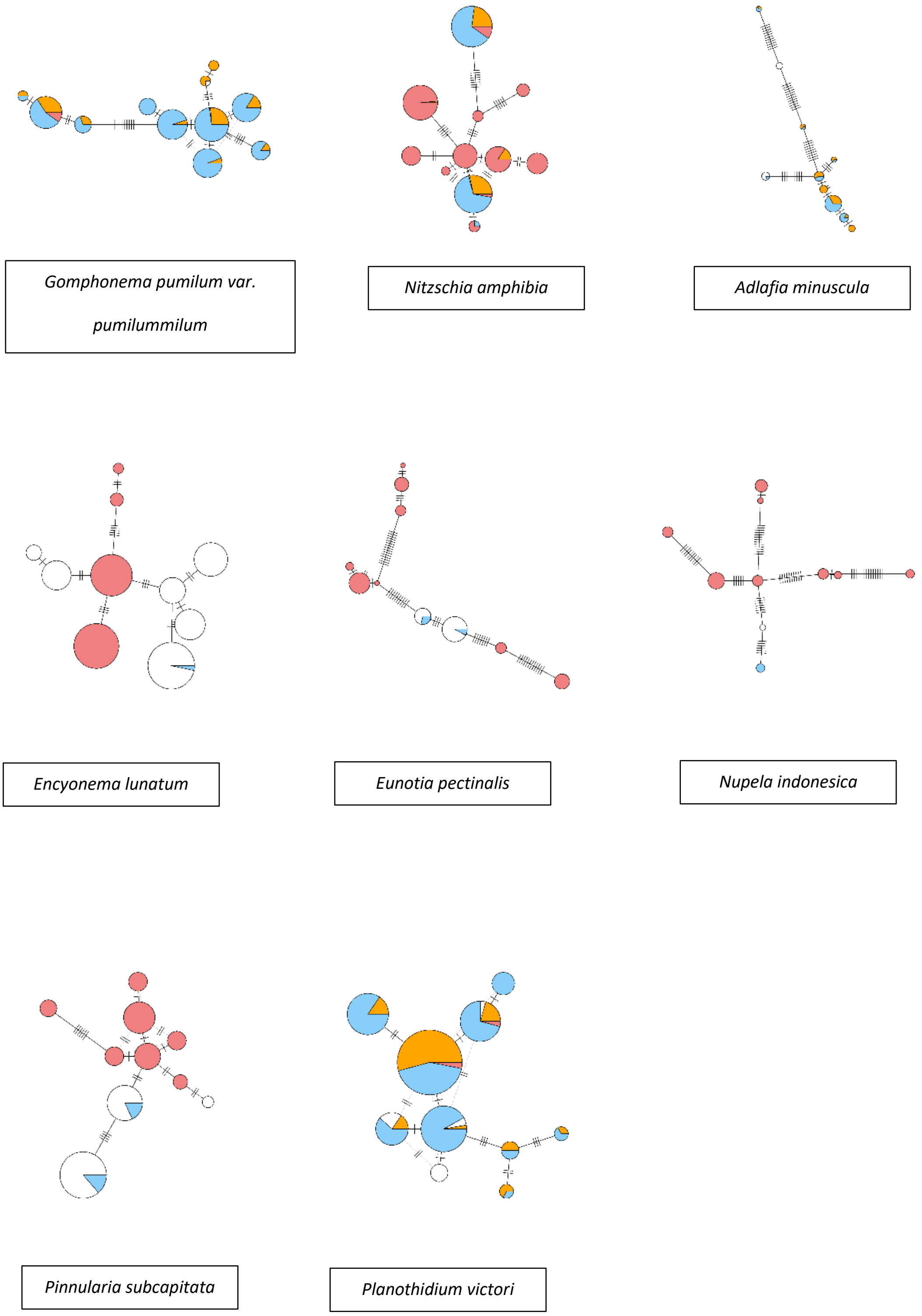

Figure S6. Haplotype network of other 32 species (see Fig. 5 of the manuscript), network performed from ASVs within each species and their read numbers presented in each climate zones. In a haplotype network, each pie chart corresponds to an ASVs, with the diameter indicating the number of reads in the studied area and the colours indicating its presence in a climate zone. For each edge connecting pie-charts, length and number of lines crossing it correspond to the number of nucleotide differences between ASVs.
