# Supplementary material for: Can genetic diversity in microalgae species be explained by climate: an overview of metabarcoding with diatoms
Source: ISME Commun. 2025 Sep 26;5(1):ycaf171. doi: 10.1093/ismeco/ycaf171 (PMC12527276; doi:10.1093/ismeco/ycaf171)
Supplement: Kulas_et_al_supplementary_information_ycaf171 [file kulas_et_al_supplementary_information_ycaf171.pdf]

Supporting information

Can genetic diversity in microalgae species be explained by climate: an overview of metabarcoding with diatoms

Short title: Diatoms genetic diversity

Antonija Kulaš[1*], Clarisse Lemonnier[2], Benjamin Alric[2,3], Maria Kahlert[4], Rosa Trobajo[5], Marija Gligora Udovič[1] & Frédéric Rimet[2,3]

[1]University of Zagreb, Faculty of Science, Department of Biology, HR-10000 Zagreb, Croatia

[2]UMR Carrtel, INRAE, Université Savoie Mont-Blanc, F-74200 Thonon les Bains, France

[3]Pôle R&D Ecosystèmes Lacustres (ECLA), F-13100 Aix-en-Provence, France

[4]Department of Aquatic Sciences and Assessment, Swedish University of Agricultural Sciences, S-750 07 Uppsala, Sweden

[5]Institute of Agrifood Research and Technology (IRTA), Marine and Continental Waters, E-43540 La Ràpita, Catalunya, Spain

Corresponding author: University of Zagreb, Faculty of Science, Department of Biology, Horvatovac 102a, HR-10000 Zagreb, Croatia. E-mail: antonija.kulas@biol.pmf.hr

Contents

Supplementary material 1 - Climate zone characterisation and evaluation of their robustness for the sampled sites.

Supplementary material 2 - Table of metadata and additional information of whole data set.

Supplementary material 3 - ASV table with number of reads across the whole dataset after bioinformatic processing.

Supplementary material 4 - Table with the DNA sequence and taxonomy assignment of the 3,302 ASVs.

Supplementary material 5 - ASV and Species Distribution Across Climate Zones: Rarefaction vs. TSS.

Supplementary material 6 - The distribution of diatom species based on their average abundance, site occupancy (occurrence), the number of ASVs (nASV) detected for each species, and their occurrence across each climate zone.

Supplementary material 7 - Heatmap of null model ratio for phylogenetic indices NRI and NTI for 36 species.

Supplementary material 8 - Haplotype networks of other 32 species, network performed from ASVs within each species and their read numbers presented in each climate zones.

Supplementary material 9 - List of 36 species, on which phylogenetic analyses were conducted.

Supplementary material 10 - Results of NRI and NTI phylogenetic indices, including clustering based on the average frequency of significant statistical tests for phylogenetic signal across climate zones.
